# The behaviour of *myo*-inositol hexakisphosphate in the presence of magnesium(II) and calcium(II): Protein-free soluble Ins*P*_6_ is limited to 49 μM under cytosolic/nuclear conditions

**DOI:** 10.1016/j.jinorgbio.2006.06.016

**Published:** 2006-11

**Authors:** Nicolás Veiga, Julia Torres, Sixto Domínguez, Alfredo Mederos, Robin F. Irvine, Alvaro Díaz, Carlos Kremer

**Affiliations:** a Cátedra de Química Inorgánica, Departamento Estrella Campos, Facultad de Química, Universidad de la República, Montevideo, Uruguay; b Departamento de Química Inorgánica, Universidad de La Laguna, Tenerife, Canary Islands, Spain; c Department of Pharmacology, University of Cambridge, Cambridge, UK; d Cátedra de Inmunología, Facultad de Química/Ciencias, Universidad de la República, Montevideo, Uruguay

**Keywords:** Bioinorganic chemistry, Calcium, Magnesium, Inositol polyphosphate

## Abstract

Progress in the biology of *myo*-inositol hexakisphosphate (Ins*P*_6_) has been delayed by the lack of a quantitative description of its multiple interactions with divalent cations. Our recent initial description of these [J. Torres, S. Dominguez, M.F. Cerda, G. Obal, A. Mederos, R.F. Irvine, A. Diaz, C. Kremer, J. Inorg. Biochem. 99 (2005) 828–840] predicted that under cytosolic/nuclear conditions, protein-free soluble Ins*P*_6_ occurs as Mg_5_(H_2_L), a neutral complex that exists thanks to a significant, but undefined, window of solubility displayed by solid Mg_5_(H_2_L) · 22H_2_O (L is fully deprotonated Ins*P*_6_). Here we complete the description of the Ins*P*_6_–Mg^2+^–Ca^2+^ system, defining the solubilities of the Mg^2+^ and Ca^2+^ (Ca_5_(H_2_L) · 16H_2_O) solids in terms of *K*_s0_ = [M^2+^]^5^[H_2_L^10−^], with p*K*_s0_ = 32.93 for M = Mg and p*K*_s0_ = 39.3 for M = Ca. The concentration of soluble Mg_5_(H_2_L) at 37 °C and *I* = 0.15 M NaClO_4_ is limited to 49 μM, yet Ins*P*_6_ in mammalian cells may reach 100 μM. Any cytosolic/nuclear Ins*P*_6_ in excess of 49 μM must be protein- or membrane-bound, or as solid Mg_5_(H_2_L) · 22H_2_O, and any extracellular Ins*P*_6_ (e.g. in plasma) is surely protein-bound.

## 1 Introduction

*myo*-Inositol hexakisphosphate (Ins*P*_6_, derived from inositol hexaphosphoric acid C_6_H_18_O_24_P_6_, abbreviated in this work as H_12_L) is an ubiquitous and abundant metabolite in eukaryotic cells (reviewed in [1–3]). It is the precursor of at least two inositol pyrophosphates; these less abundant, fast-turnover metabolites, have been assigned a variety of functions including the regulation of membrane trafficking, telomere length, and membrane–protein interactions involved in chemotaxis (reviewed in [4]). However, the functions of Ins*P*_6_ itself are still poorly understood. In mammalian cells, it is a necessary cofactor for the repair of double-stranded breaks in DNA, through the binding of the Ku component of DNA-PK, and is necessary for RNA editing events, through its binding of some of the adenosine deaminase enzymes [5–8]. In yeasts, the compound is necessary for mRNA export from the nucleus, through as yet undefined protein targets [9–11]. In addition, Ins*P*_6_ may contribute to localise PDK-1 to the cytosol, by competing for the enzyme’s PH domain with plasma membrane phosphoinositides [12]. Ins*P*_6_ concentrations in animal and yeast cells are not thought to undergo acute fluctuations under normal physiological conditions: the three functions mentioned above are apparently fulfilled by basal concentrations of Ins*P*_6_. In plant cells, Ins*P*_6_ levels can have significant signalling roles by regulating K^+^ and Ca^2+^ fluxes [13,14]. In addition, a major function of Ins*P*_6_ in plants is to give rise to deposits for the storage of phosphorus and metal cations, as further discussed below.

In addition to the apparently unconnected set of roles mentioned above, Ins*P*_6_ has been assigned a bewildering number of other biological functions as well as pharmacological actions. This has been critically reviewed by Shears who pointed out that a number of the proposals in the literature may arise from experimental artefacts [2]. Indeed, it is easy to run into artefacts when carrying out biological experiments with Ins*P*_6_. This stems from: (i) the compound being abundant, and so including high concentrations of it in experiments is initially justified, and (ii) its chemistry of interaction with divalent cations being complicated, non-intuitive, and so far poorly described. This chemistry encompasses solution complexation and precipitation reactions. Complexation can deplete solutions of divalent cations, while precipitation (easily unnoticed when working with small volumes) can not only deplete divalent cations, but also acidify the solutions and interfere with assays per se.

Equally seriously, the lack of understanding of Ins*P*_6_ chemistry means that the physical and chemical forms of the compound in cells are still partially obscure. This problem is compounded by the questions about the cellular distribution of Ins*P*_6_. Most Ins*P*_6_ in mammalian cells is thought to be cytosolic and/or nuclear [15]. In addition, in plants [16–18], and in a particular (invertebrate) animal system [19,20], the compound occurs in vesicular/vacuolar and/or extracellular compartments, there appearing as “phytate deposits”, i.e. Ins*P*_6_ salts with monovalent, and mostly divalent, cations. Speculations on the status of Ins*P*_6_ in mammalian cells encompass the possible existence of vesicular pools as well as of insoluble deposits in cytosol (see for example [15,21]). In addition, dietary Ins*P*_6_ has been shown to be absorbed in the intestine, circulate in plasma, and to be excreted in urine (reviewed in [22]).

The chemistry of Ins*P*_6_ in solution in the presence of divalent cations has been addressed in several works (see in particular [23,24]). However, this chemistry was only comprehensively described, and its biological consequences directly inferred, in our recent study [25]. A major feature of this chemistry is that Ins*P*_6_ forms high-affinity complexes of 1:1 stoichiometry with divalent (and trivalent) cations; this is the dominant behaviour when Ins*P*_6_ is in molar excess with respect to cation(s). A further, biologically significant, feature of the solution chemistry is that Ins*P*_6_ also forms, in the presence of molar excess of Mg^2+^, the neutral soluble species Mg_5_(H_2_L) (where L denotes fully deprotonated Ins*P*_6_). Under cytosolic or nuclear conditions, all soluble Ins*P*_6_ not bound to proteins or other organic components is predicted to be found in this form (Ins*P*_6_ binds at least certain proteins bearing very basic sites in Mg^2+^-free form [8]). Although we verified experimentally that relevant concentrations of Mg_5_(H_2_L) could exist under cytosolic/nuclear conditions, the species is only sparingly soluble; in fact, isolation of the corresponding solid, Mg_5_(H_2_L) · 22H_2_O, was straightforward [25]. The significant (though small) solubility of Mg_5_(H_2_L) is a peculiarity, not displayed by other divalent cations, Ca^2+^ included: the dominant aspect of Ins*P*_6_ chemistry under molar excess of the divalent (or trivalent) cations is the formation of solids.

The limited solubility of its presumed cytosolic form Mg_5_(H_2_L), plus the proven formation of solids by Ins*P*_6_ in certain biological contexts (and the possibility of this taking place in others), call for a quantitative description of the solubility behaviour of this metabolite. The experimental problems (described above) arising from not being able to predict this behaviour also call for such a study, and this in turn requires the stoichiometries of the solids to be known. Stoichiometries reported for the divalent metal solids of Ins*P*_6_ range from 4:1 to 6:1 (metal:Ins*P*_6_ ratios); the 5:1 ratio, as we found for the Mg^2+^ solid [25], is the one reported most often [23,24,26–29]. The precipitation equilibria are tied to the complexation and protonation equilibria in solution, so the quantitative description of these [25] is also needed to describe the solubility behaviour. In the present work, we have determined the stoichiometry of the Ca^2+^ salt of Ins*P*_6_; together with the data in our previous work, this allowed us to translate analytical measurements of total Ca and Mg at equilibrium with the solid phytates into solubility product constants for the two salts. Therefore a full description of the Ins*P*_6_–Ca^2+^–Mg^2+^ system has resulted. The data put an upper limit to the concentration of Ins*P*_6_ that can exist in cytosol or nucleus of mammalian cells in protein-free, soluble form, a limit that is within the range of available estimates of the total Ins*P*_6_ concentration in cells. Our data also indicate that protein-free extracellular Ins*P*_6_ cannot exist in solution. In addition, we have been able to summarise the complex and non-intuitive solubility behaviour of Ins*P*_6_ in a series of plots that indicate clearly whether total, partial or nil solubility is to be expected across a wide range of conditions.

## 2 Experimental

### 2.1 Chemicals

All common laboratory chemicals were reagent grade, purchased from commercial sources and used without further purification. CaCl_2_ · 2H_2_O, and MgCl_2_ · 6H_2_O, were used as metal sources. Phytate solutions for the synthesis of the complexes were prepared by dilution of a phytic acid solution in water (40 wt.%; Aldrich). Solutions were used immediately after preparation. Ultrapure water obtained from a Millipore-MilliQ plus system was used throughout this work.

### 2.2 Infrared spectroscopy, thermal analysis, and elemental analysis

Infrared spectroscopy was carried out on a Bomen FT-IR spectrophotometer, with samples present as KBr (1%) pellets. Thermal analysis was performed on a Shimadzu DTA-50, TGA-50 instrument with a TA 50I interface, using a platinum cell and nitrogen atmosphere. Experimental conditions were 1 °C min^−1^ temperature ramp rate and 50 mL min^−1^ nitrogen flow rate.

Elemental analysis (C, H) was performed on a Carlo Erba EA 1108 instrument. Na and K were determined by atomic absorption spectroscopy on a Perkin–Elmer 5000 instrument. Ca content was determined gravimetrically as CaC_2_O_4_ as follows: calcium phytate was dissolved in 2 M HCl and 100% excess of H_2_C_2_O_4_ · 2H_2_O was added and the pH of the solution was raised with NH_4_OH (7.4 M) up to 1.5–3.0. Calcium oxalate then precipitated, and was washed with water (2 × 5 mL), centrifugued, and dried at 70 °C for 12 h.

### 2.3 Synthesis of [Mg_5_(H_2_L)] · 22H_2_O and [Ca_5_(H_2_L)] · 16H_2_O

The magnesium compound was prepared as previously reported [25]. Preparation of [Ca_5_(H_2_L)] · 16H_2_O followed a similar procedure. An aqueous solution of Ins*P*_6_ (0.01 M) was prepared and its pH adjusted to 10–11 by addition of LiOH (1 M). To this solution (30 mL; 0.3 mmol), CaCl_2_ · 2H_2_O (0.22 g; 1.5 mmol) dissolved in the minimum amount of water was added. A white solid immediately appeared, which was separated by centrifugation, thoroughly washed with water (3 × 10 mL), and dried with ethanol (2 × 10 mL). Yield was 37% (126 mg). Elemental analysis calcd (%) for Ca_5_C_6_H_40_O_40_P_6_ (1138.59): C 6.3, H 3.5, Ca 17.6; found C 6.4, H 3.3, Ca 17.7. Thermal analysis agreed with the proposed formula: 25.3% weight loss corresponding to the elimination of water, compared with a calculated value of 25.6%. IR (KBr pellets) *ν* = 3447 (*ν*_O–H_), 1114 (*ν*_P–O_), 545 (*ρ*_w_, H_2_O) cm^−1^.

### 2.4 Solubility measurements

Solubility measurements were carried out at constant ionic strength *I* = 0.15 M NaClO_4_, and 37.0 °C. Approximately 50 mg of the compound (Mg or Ca phytate) was suspended in 0.15 M aqueous NaClO_4_ (Mg, 20.0 mL; Ca, 15.0 mL). Known amounts of HCl were added, so as to reach equilibrium points corresponding to measurable amounts of metal in solution (Table 1 ). Each mixture was kept in a glass jacketed cell under continuous stirring until measured pH was constant (ca. one week). After the equilibrium was reached, excess solid was filtered out (Macherey–Nagel MN 640 m paper), and the metal concentration was determined in the supernatant. Mg was determined volumetrically according to standard techniques [30]. Ca was determined as described in Section 2.2. With these M^2+^ concentration values, and assuming a 5:1:2 stoichiometry (M^2+^:Ins*P*_6_:H^+^), total amounts of Ins*P*_6_ were calculated. Then total concentrations of M^2+^, Ins*P*_6_ and H^+^ were used as inputs in the HySS software [34] to determine the equilibrium concentrations of (free) M^2+^ and H_2_L^10−^, which define the *K*_s0_. In this calculation, the complete set of equilibria involved [25] was taken into account. At least four independent determinations were performed for each metal.

## 3 Results and discussion

### 3.1 Stoichiometry of calcium and magnesium phytates

As mentioned, previous works dealing with the interaction of M^2+^ ions with Ins*P*_6_ under metal excess report the formation of very insoluble compounds having M^2+^:Ins*P*_6_ ratios of 4:1, 5:1 or 6:1 [23–29]. The solids contain large amounts of crystallization water, which hampers their full characterization. An additional feature reported is the presence of mixed salts containing two cations (for example Ca and Na, Ca and Zn, etc.) [31–34] or two anions (Cl^−^ and Ins*P*_6_) [23].

We previously prepared and characterized by several techniques the salt [Mg_5_(H_2_L)] · 22H_2_O [25]. In our hands, the calcium analogue formed under straightforward conditions fitted perfectly this same (5:1) stoichiometric ratio. The solid incorporated 16 water molecules, thus responding to the formula [Ca_5_(H_2_L)] · 16H_2_O. The presence of a large amount of crystallized solvent was qualitatively evident from the strong and broad absorption in the IR spectrum at 3447 cm^−1^. Quantification of water content by thermogravimetric analysis showed that the sixteen water molecules were lost across a wide temperature range, namely between 50 and 210 °C.

To assess whether the Mg^2+^ and Ca^2+^ solids can change their compositions in the presence of Na^+^ or K^+^, we performed the syntheses as described in Section 2 but in media containing either 0.15 M NaClO_4_ or 0.15 M KCl. The solids so obtained agreed perfectly with the expected formula $[M_{5}^{\mathrm{II}}(H_{2}L)]\cdot xH_{2}O$. Na or K determinations by atomic absorption demonstrated that only residual amounts (i.e. less than 0.1% molar ratio with respect to M^2+^) of the alkaline cations were present, probably as occluded chloride salts not eliminated during the washing procedure. Thus the formation of mixed cation compounds with fixed M^2+^/Na or M^2+^/K stoichiometries can be ruled out under these experimental conditions.

### 3.2 Solubility product constants of calcium and magnesium phytates

Mg and Ca phytates are only sparingly soluble in water. Given their stoichiometries, the solubilities of these solids will be governed by solubility product constants with the form: *K*_s0_ = [M^2+^]^5^[H_2_L^10−^]; the constant *K*_s0_ depends only on the temperature. It is easy to appreciate that precipitation of Mg or Ca phytate depends strongly on the concentration of free Ca^2+^ or Mg^2+^ in the system. Moreover, it further depends on the concentration of di-protonated phytate, H_2_L^10−^. This is very much a minority species for soluble Ins*P*_6_ under most conditions, the dominant species being the less highly charged ones, in which the anion is associated with more protons, and/or monovalent and/or divalent cations [25]. This notwithstanding, with other conditions fixed, the concentration of H_2_L^10−^ (a highly deprotonated species) can be expected to increase as pH is increased. This rationalises the straightforward observation that phytates are more soluble under acidic than under basic conditions. A closely related point is that the precipitation of phytates will tend to acidify the solutions in which it takes place. For example, the dominant Ins*P*_6_ species in solution in the absence of divalent cations and at neutral pH have three and four protons [25]. Addition of sufficient calcium or magnesium removes, through precipitation, H_2_L^10−^ from solution. This species is in turn replenished at the expense of the more highly protonated forms of soluble Ins*P*_6_, with the release of protons into solution.

Determination of the *K*_s0_ values obviously requires measuring sets of values for the concentrations of H_2_L^10−^ and of free metal at equilibrium with the solids. These can in turn be calculated from the straightforward analytical data by means of an appropriate software such as HySS [35] fed with the complete set of equilibrium constants for the protonation and complexation equilibria [25]. We thus equilibrated the solid phytates with unbuffered, M^2+^-free, dilute acid at 37.0 °C and under physiological ionic strength. Then total Ca or Mg in solution were determined, and the amounts of Ins*P*_6_ that went into solution calculated as 1/5 the former figures. From the total concentration of Ins*P*_6_, M^2+^, and total exchangeable protons present in the solution, the HySS software calculated concentrations of H_2_L^10−^ and of free metal, yielding in turn estimations for p*K*_s0_ (i.e. −log *K*_s0_). The results shown in Table 1 can be summarised in the solubility products:

$$\begin{array}{cc} & [{\mathrm{Mg}}_{5}(H_{2}L)]↔5{\mathrm{Mg}}^{2+}+H_{2}L^{10-}\text{,}\;pK_{s0}=32.93(6)\text{,}\\ & [{\mathrm{Ca}}_{5}(H_{2}L)]↔5{\mathrm{Ca}}^{2+}+H_{2}L^{10-}\text{,}\;pK_{s0}=39.3(4)\text{,} \end{array}$$

which are valid at *I* = 0.15 M NaClO_4_ and 37.0 °C.

The higher p*K*_s0_ value for Ca reflects the lower solubility of Ca phytate in comparison to its Mg analogue. With these values in hand, it was possible to calculate the solubility of both compounds in water at different pH values (under physiological ionic strength and in the absence of added Ca^2+^ or Mg^2+^), as shown in Table 2 .

### 3.3 A complete description of the behaviour of the Ins*P*_6_ in the presence of Ca^2+^ and Mg^2+^

The p*K*_s0_ values for the solid phytates, together with the protonation and Ca and Mg complexation constants in solution previously reported by us [25] allow a complete description of the behaviour of Ins*P*_6_ in the presence of Ca^2+^ and/or Mg^2+^. Since this is a system comprising 22 equilibrium equations, it can only be handled by specialised software such as HySS [35].

**Fig. 1:**
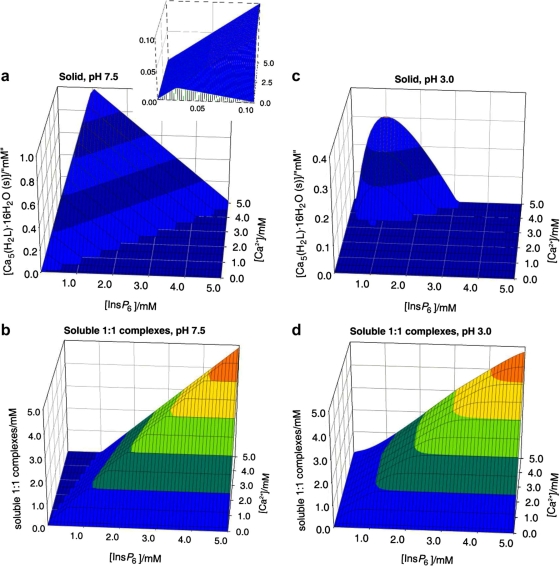
Behaviour of Ins*P*_6_ in the presence of calcium. The graphs show the predicted abundances of solid calcium phytate (a, c) and of the sum of the different soluble, 1:1, Ca:Ins*P*_6_ complexes (b, d), plotted against total concentrations of Ins*P*_6_ (0–5 mM) and Ca^2+^ (0.1–5 mM). The inset in (a) shows a “zoom-in” of the range up to 0.1 mM total Ins*P*_6_. Predictions are drawn for pH 7.5 (a, b) and pH 3.0 (c, d), always in 0.15 M NaClO_4_ and 37.0 °C. Note that the abundance scales in different parts are different.

Fig. 1 summarises the behaviour of Ins*P*_6_ in the presence of Ca^2+^. It can be seen that at pH 7.5 Ca^2+^ causes Ins*P*_6_ to fall out of solution even at very low concentrations of the polyphosphate. At a fixed total calcium concentration, as the total Ins*P*_6_ concentration is raised, the abundance of the solid phytate increases, reaching a maximum near to the stoichiometric (5:1) calcium:Ins*P*_6_ ratio. As Ins*P*_6_ concentration is increased beyond this point, the solid (Fig. 1a) is gradually replaced by the soluble 1:1 complexes (Fig. 1b). This is the “paradoxical” solubility behaviour of Ins*P*_6_, whereby for some concentration ranges, adding Ins*P*_6_ to a partially insoluble system causes it to become fully soluble. This behaviour can be rationalised on the basis of the excess Ins*P*_6_ acting, through the formation of the soluble 1:1 complexes, as a calcium sequestering agent. Thus in this context the addition of excess Ins*P*_6_ is analogous to the addition of EDTA (ethylenediaminetetraacetate), the system’s solubility being therefore caused by the absence of free Ca^2+^. The broad features of the Ins*P*_6_-calcium system do not vary to a great extent with pH, except for strongly acidic conditions, i.e. pH 3 and below. For these acidic conditions, the range of dominance of solid phytate shrinks, with a concomitant increase in the dominance range of the 1:1 complexes (Fig. 1c and d). This is the consequence of the linked equilibria established by soluble Ins*P*_6_ being shifted away from H_2_L^10−^ towards the more highly protonated species as well as the 1:1 complexes that these can form with Ca^2+^.

**Fig. 2:**
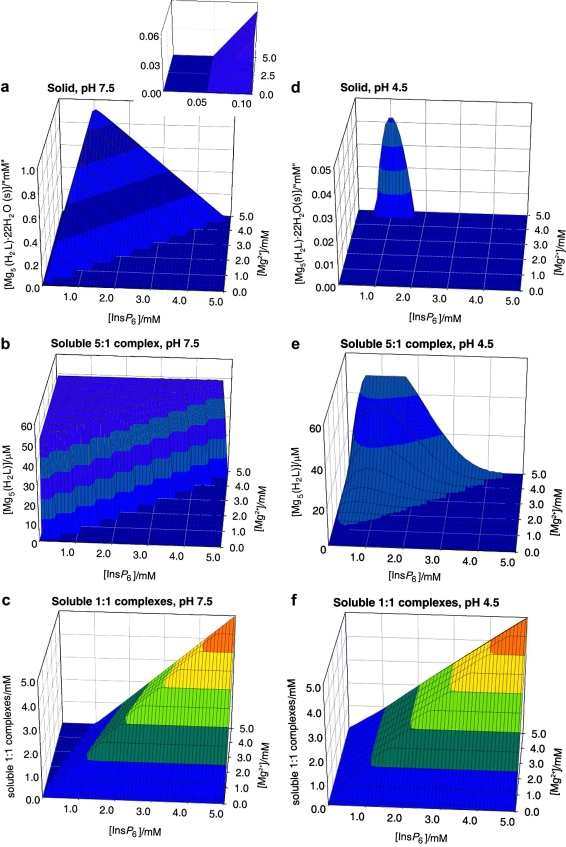
Behaviour of Ins*P*_6_ in the presence of magnesium. The graphs show the predicted abundances of solid magnesium phytate (a, d), of the soluble 5:1 complex (b, e), and of the sum of the different soluble 1:1 complexes (c, f), all plotted against total concentrations of Ins*P*_6_ (0–5 mM) and Mg^2+^ (0.1–5 mM). Predictions are drawn for pH 7.5 (a–c) and pH 4.5 (d–f), always in 0.15 M NaClO_4_ and 37.0 °C. The inset in (a) shows a “zoom-in” of the range up to 0.1 mM total Ins*P*_6_. Note that the abundance scales for (b) and (e) are different from the rest.

The behaviour in the presence of Mg^2+^ is slightly more complex. This is due to the 5:1 species having a significant window of solubility, a feature that is undetectable in the case of Ca^2+^ [25]. Thus under Mg:Ins*P*_6_ excess, the dominant species in solution is the neutral 5:1 complex. However, beyond a certain concentration, this species precipitates, as [Mg_5_(H_2_L)] · 22H_2_O. Hence, to summarise the behaviour of the Ins*P*_6_–Mg^2+^ system, three major forms need to be considered: the solid, the soluble 5:1 species, and the soluble 1:1 complexes (Fig. 2 ). With respect to the solid and the soluble 1:1 complexes, the overall behaviour is similar to that described previously for Ca^2+^ (Fig. 2a and c). However, the lower p*K*_s0_ of the system is reflected in a significant region of full solubility even under conditions of metal excess (compare Fig. 2a, inset with Fig. 1a, inset); in this window, virtually all of the Ins*P*_6_ is present as the soluble neutral 5:1 species (Fig. 2b). Interestingly, the concentration of this soluble 5:1 complex has an upper limit at 49 μM, as figure that is fixed, irrespective of total Ins*P*_6_ concentration, size of the Mg^2+^ excess, or pH. Mathematically, this is the consequence of the fact that multiplying the expression for the equilibrium constant for the formation of the soluble 5:1 complex [25]

$$K=[{\mathrm{Mg}}_{5}(H_{2}L)]/({\left[{\mathrm{Mg}}^{2+}\right]}^{5}[H_{2}L^{10-}])$$

by the expression for the solubility product constant

$$K_{s0}={\left[{\mathrm{Mg}}^{2+}\right]}^{5}[H_{2}L^{10-}]$$

yields

$$K\cdot K_{s0}=[{\mathrm{Mg}}_{5}{\left(H_{2}L\right)}_{\left(\mathrm{aq}\right)}]=4.9\times 10^{-5}\text{.}$$

In other words, the concentration of soluble pentamagnesium species at equilibrium with solid magnesium phytate is a constant at a given temperature. Under conditions of Mg^2+^ excess, increasing total Ins*P*_6_ beyond 49 μM always means that the concentration of the soluble pentamagnesium complex also breaks the 49 μM barrier, and hence solid magnesium phytate starts to accumulate (Fig. 2a and b). The overall behaviour of the Mg^2+^–Ins*P*_6_ system is fairly invariant for pH values above 6. However, at acidic pH values the ranges of formation of the soluble and solid 5:1 species shrink. This behaviour is similar to that of the Ca^2+^ system but more pronounced, being very marked already at pH 4.5 (Fig. 2d–f).

**Fig. 3:**
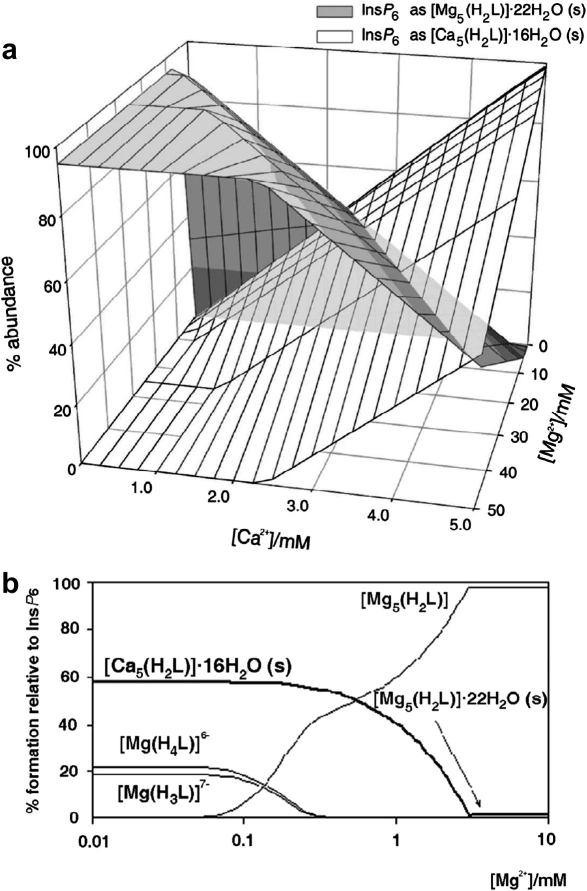
Behaviour of Ins*P*_6_ in the presence of calcium and magnesium. In (a), the predicted abundance of solid calcium and magnesium phytates is plotted, for a fixed total Ins*P*_6_ concentration of 1 mM and pH 7.5, against total Ca^2+^ and Mg^2+^ concentrations. The plot in (b) shows the behaviour of the system at a total Ins*P*_6_ concentration (50 μM) near the solubility limit of magnesium phytate, in the presence of 150 μM Ca^2+^, also at pH 7.5; note that the presence of Mg^2+^ confers solubility to an otherwise insoluble system. Predictions are for *I* = 0.15 M NaClO_4_ and 37.0 °C. Note that “(s)” in graph labels stands for “solid”.

The behaviour of the system with both Ca^2+^ and Mg^2+^ present is broadly similar to the single-metal ones just described, as long as the concentrations of the two cations are considered together. As previously described, molar excesses of cation over Ins*P*_6_ are accompanied by precipitation, while molar excesses of Ins*P*_6_ result in soluble 1:1 complexes. Within the range of formation of the solids, the more insoluble calcium phytate dominates over its magnesium counterpart. Notwithstanding, as long as the Ca^2+^:Ins*P*_6_ ratio is less than 5, and Mg^2+^ is abundant, magnesium phytate does precipitate together with calcium phytate (Fig. 3 a). For Ins*P*_6_ concentrations below 49 μM and similar concentrations of Ca^2+^, the presence of excess Mg^2+^ actually confers solubility to the system, as Ins*P*_6_ is drawn towards the soluble pentamagnesium complex (Fig. 3b).

### 3.4 Predictions for Ins*P*_6_ under cytosolic and nuclear conditions

The chemical data presented above make it possible to predict the speciation of Ins*P*_6_ under biological conditions. The most relevant of these conditions are those of cytosol and nucleus, in which most or all of the Ins*P*_6_ in typical animal cells is probably present [15]. In our previous paper [25], to simulate cytosolic/nuclear conditions we had set the prediction conditions at pH 7.4 [36] and 0.5 mM free Mg^2+^ [37]; Ca^2+^ signals were taken into account by including total Ca^2+^ concentrations of up to 10 μM (reviewed in [38]). Those calculations indicated that all Ins*P*_6_ not bound to protein and/or membranes existed as the pentamagnesium complex [25]. At the time we did not have the solubility product constants, and so we stated that the validity of our predictions was restricted to the solubility range of magnesium phytate. When predictions including the precipitation equilibria are run for the set of conditions detailed above and Ins*P*_6_ concentrations between 1 and 100 μM, the following results are obtained: (i) there is no association with Ca^2+^, whether in solution or in the solid phase; (ii) up to a total concentration of 49 μM, Ins*P*_6_ is present exclusively as the soluble pentamagnesium complex; (iii) any Ins*P*_6_ in excess of 49 μM, is present as solid magnesium phytate. Therefore our previous predictions are confirmed, but a complication arises as a result of the limited solubility of Mg_5_(H_2_L).

Estimations of total Ins*P*_6_ concentrations in mammalian cells are mostly in the 10–60 μM range, with data from some cell lines going up to 105 μM [39–45]. As these estimations do not take cell compartmentalisation into account, actual concentrations are probably higher. It is conceivable that Ins*P*_6_ in cells is at equilibrium with its solid magnesium salt – note that massive amounts of solid magnesium Ins*P*_6_ exist in a peculiar biological system, namely within specialised cells of the dispersal larva of the mesozoan *Dicyema typus* [46]. However, we envisage as the more likely possibility that the Ins*P*_6_ pool available for the formation of Mg_5_(H_2_L) is kept below 49 μM in typical eukaryotic cells. From this standpoint, any Ins*P*_6_ in excess of this figure would be bound to cellular components such as proteins and/or cell membranes [47]. Polyamines are unlikely to contribute substantially to the Ins*P*_6_-binding capacity of the cell. This reasoning is based on the facts that: (i) once association with nucleic acids and nucleotides is taken into account, the remaining pools of spermidine and spermine are relatively small [48], and (ii) Ins*P*_6_ affinity constants reported for polyamines are not large enough for effective competition with the more abundant Mg^2+^, except in the case of the tri-protonated forms of polyamines, which represent only a minor proportion at physiological pH [49].

In summary, our data suggest that Ins*P*_6_ in the cytosol and nucleus of mammalian cells is close to the saturation of its solubility. Therefore the manipulation of cells so as to increase their Ins*P*_6_ concentrations to a significant extent (e.g. [50–52]) probably causes the intracellular precipitation of magnesium phytate. It is worth commenting at this point that although, as mentioned, Ins*P*_6_ has a second range of solubility at concentrations in molar excess with respect to total Mg^2+^ plus Ca^2+^, these conditions are non-physiological, as they entail the complete absence of free divalent cations.

The case of slime molds deserves a special mention is this context. The overall concentration of Ins*P*_6_ in *Dictyostelium discoideum* amoebae has been estimated at 0.7 mM [53]; no information on the intracellular distribution of the compound is available. Our data predict that whatever its localisation, most of this Ins*P*_6_ must be present in a physicochemical form other than the soluble pentamagnesium species. It seems likely that Ins*P*_6_ in *D. discoideum* may form deposits in so-called “mass-dense granules” or “polyphosphate bodies”; these are acidic organelles rich in P, Mg and Ca, which are now known to be intimately related to the contractile vacuoles [54–57]. Phosphorus in these granules has been ascribed to inorganic pyrophosphate and polyphosphates [54,55]; however, the presence of Ins*P*_6_ in them, suggested by Schlatterer et al. [56], has not yet been assessed. Electron-dense granules ascribed to polyphosphate deposits have been also described, though in lesser abundance, in the cytosol, nucleus and mitochondria of *D. discoideum* [55]. As the overall Ca^2+^:Ins*P*_6_ molar ratio in *D. discoideum* amoebae is less than 1 [53], phytate deposits can be expected to be formed mostly by magnesium phytate, irrespective of their localisation. If indeed localised in the acidic “mass-dense granule” vacuoles, the Ins*P*_6_ solid(s) would be at equilibrium with significant concentrations of the soluble compound. This would both make the Ins*P*_6_ pool a metabolically active one, and allow the compartment’s osmolarity to be regulated through the pH-dependent solubilisation/precipitation of Ins*P*_6_.

### 3.5 Predictions for Ins*P*_6_ in vesicular compartments and under extracellular conditions

The p*K*_s0_ of calcium phytate is very high. Therefore the set of equilibria described in this work and the previous one [25] allow no soluble Ins*P*_6_ under conditions that include physiological pH and extracellular concentrations of free Ca^2+^ and Mg^2+^. Being present at concentrations similar to those of Ca^2+^, Mg^2+^ has no impact on the system, so Ins*P*_6_ is predicted to be entirely in the form of solid calcium phytate. This is basically the case of the only known system that displays extracellular accumulation of Ins*P*_6_, namely the larva of the cestode parasite *Echinococcus granulosus* [19,20]. However, a soluble extracellular Ins*P*_6_ pool has also been suggested to exist in mammals: although widely varying according to diet, Ins*P*_6_ levels in human or rat plasma have been reported to reach around 0.5 μM [58,59]. Our data predict that this extracellular pool will be associated with carrier proteins. A further important consequence of our results is that experiments in which exogenous Ins*P*_6_ is added to cells in culture (see for example [60–62], out of an extensive literature) have to be examined with caution. The two obvious possibilities are: i) cells are being fed particulate calcium phytate, with concomitant lowering of the Ca^2+^ concentration, and possibly also pH, of the medium (when using sub-millimolar Ins*P*_6_ concentrations), and ii) the medium is being depleted of free Ca^2+^ and Mg^2+^ through complexation in solution (when millimolar Ins*P*_6_ doses are used). A third possibility (only likely to apply for Ins*P*_6_ concentrations in the μM range) is that the medium may contain proteins with sufficient Ins*P*_6_-binding capacity as to keep it soluble.

### 3.6 Qualitative predictions relevant to the nutritional problems associated with Ins*P*_6_

Phytate has long been recognised to interfere with the absorption of Zn, Fe, and Ca in the gut (reviewed in [63]). Our data provide a rationale for explaining the effect on Ca, and possibly on the remaining metals mentioned. The bulk of the phytate counterions in cereals is accounted for by Mg and K [17,18]. Under highly acidic digestion conditions in the stomach (pH 1–2), these Ins*P*_6_ salts are expected to dissolve. Corn grain for example contains approximately 4 mol of Mg per mol of Ins*P*_6_ (Max Tate, University of Adelaide, personal communication, based on data in [18]); therefore almost any Ca or additional Mg in the meal would put the system under conditions of divalent metal excess with respect to Ins*P*_6_. In consequence, under duodenal conditions (pH 6–7), Ins*P*_6_ is predicted to (re-)precipitate. Our data predict that this precipitation will take virtually all Ca^2+^ present (plus enough Mg^2+^ as to complete the 5:1 M^2+^:Ins*P*_6_ ratio). In other words, the acidification-neutralisation cycle in vertebrate digestion allows cations that form relatively more soluble solids with phytate to be replaced by those that form less soluble solids. Thus it may be precipitation, and not complexation in solution, that is the most likely reason for Ca malabsorption by phytate. Although p*K*_s0_ values for the corresponding solids are not yet available, the same mechanism probably applies to malabsorption of Fe and Zn.

### 3.7 A “user’s guide” for the experimentation with Ins*P*_6_

The data in this paper provide a few simple “rules of thumb” to keep experiments involving Ins*P*_6_ within reasonably physiological conditions. When mimicking cytosolic/nuclear conditions, total Mg^2+^ should be set at a concentration exceeding (by at least 0.5 mM) five times the concentration of Ins*P*_6_; obviously, additional Mg^2+^ must be included when in the presence of further Mg^2+^ complexating agents such as ATP. In addition, total Ins*P*_6_ concentrations must be kept below 49 μM, unless working with particulate phytate is desired and/or a substantial Ins*P*_6_-binding capacity (in proteins or membranes) is expected. In order to imitate extracellular or vesicular system conditions, a millimolar-range excess of Ca over five times the molar amount of Ins*P*_6_ present must be included. Moreover, it must be borne in mind that Ins*P*_6_ will be present in solid form (generally as a very fine precipitate), except for the fraction of it that may be bound by proteins.

**Fig. 4:**
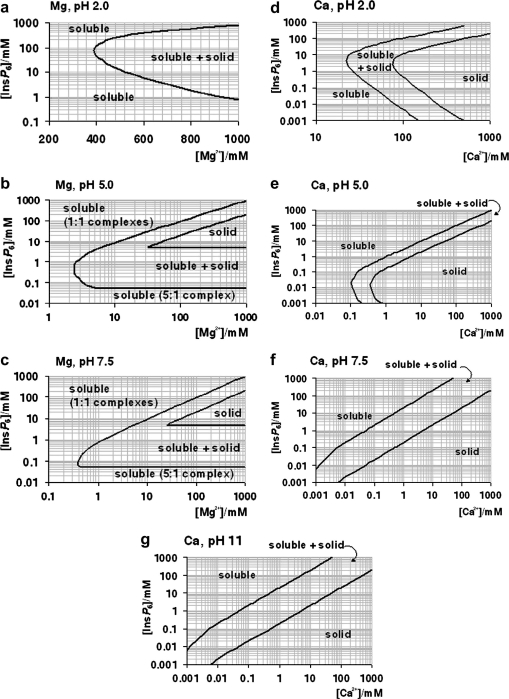
A “user’s guide” to Ins*P*_6_ in the presence of calcium and magnesium. The solubility behaviour of Ins*P*_6_, as predicted by the equilibrium equations reported in this work and in [25], in the presence of Mg^2+^ (a–c) or Ca^2+^ (d–g), is plotted for different pH values; plots for the Mg^2+^ system at basic pH values (above pH 7.5) are identical to that shown for pH 7.5. The “frontier lines” drawn correspond to conditions in which either 1% or 99% of Ins*P*_6_ present is predicted to exist as a solid. Predictions were obtained by means of the HySS software, and are valid for *I* = 0.15 M NaClO_4_ and 37.0 °C. In addition, the validity of the predictions may be limited by the potential precipitation, at high Ins*P*_6_ concentrations, of salts containing Na^+^, Na^+^/Ca^2+^ or Na^+^/Mg^2+^, which have not been studied.

Many experiments and procedures require conditions other than those mentioned above. These include the preparation of stock solutions, the extraction of Ins*P*_6_ from biological samples, experiments mimicking intestinal conditions, and assays of phytase activity. As mentioned, the multiple equilibrium constants involved can only be put to practice with the help of specialised software, and even so, with some technical difficulties. We have thus put together a series of plots that summarise the solubility behaviour of Ins*P*_6_ in the presence of Ca^2+^ and Mg^2+^ (Fig. 4 ). In these plots, the frontiers between solubility and precipitation are given in terms of total concentrations of Ins*P*_6_ and metal. Overall, for each given condition, the area of dominance of solid phytate in the plots is wedged within the region of full solubility, which encompasses both low and high Ins*P*_6_ concentrations. However, for Ca^2+^ at neutral and alkaline pH, the low-[Ins*P*_6_] solubility region does not exist, reflecting that as long as enough metal is present, even very low concentrations of phytate are insoluble. For Mg^2+^ at neutral and alkaline pH, the low-[Ins*P*_6_] solubility region corresponds to the dominance of Mg_5_(H_2_L), and it has therefore a straight-line limit at [Ins*P*_6_] = 49 μM.

The plots in Fig. 4 are given in terms of total metal ion. For neutral and alkaline pH, free cation can be calculated as total cation minus five times total Ins*P*_6_; when [M^2+^]_total_ is less than 5 × [Ins*P*_6_]_total_, free divalent cations are absent. It is important to bear in mind that in some situations it is the concentration of *free* metal ion that is fixed. For example, when Ins*P*_6_ is parenterally administered, extracellular fluids can be expected to equilibrate with respect to (free) Ca^2+^: thus once Ins*P*_6_ precipitates, the local total “concentration” of Ca^2+^ will be equal to the overall physiological [Ca^2+^]_free_ plus the five times the local Ins*P*_6_ “concentration”. Similarly, when dialysis of Ins*P*_6_ against Ca^2+^- or Mg^2+^-containing media is attempted, free metal equilibrates between the two compartments, and Ins*P*_6_ precipitation takes place.

Conditions including both Ca^2+^ and Mg^2+^ are difficult to summarise. However, a simple rule is that within dominance ranges of calcium phytate (Fig. 4d–g), the additional inclusion of Mg^2+^ will not alter the system, except under the very special set of conditions depicted in Fig. 3b. Another rule of thumb is that, at the same total divalent cation concentration, Ca^2+^–Mg^2+^ systems will be less soluble than the corresponding Mg^2+^-only systems.

Our data are derived from constants measured in the presence of 0.15 M NaClO_4_. Including K^+^ instead of Na^+^ does not change the systems significantly ([64] and our unpublished results). However, changes in ionic strength are expected to alter the systems, with higher ionic strength generally enhancing solubility and viceversa.

## Figures and Tables

**Table 1:** Determination of p*K*_s0_ for Mg and Ca phytates^a^

Cation	H^+^ added (μmol)	[M^2+^]_tot_ (mM)	[Ins*P*_6_]_tot_ (mM)	[H^+^]_tot_ (mM)	[M^2+^]_free_ (M)	[H_2_L^10−^]_free_ (M)	p*K*_s0_
Mg	4.89	0.781	0.156	0.556	4.01 × 10^−4^	1.29 × 10^−16^	32.87
	19.6	1.84	0.369	1.71	1.28 × 10^−3^	3.41 × 10^−19^	32.93
	19.6	1.83	0.366	1.70	1.28 × 10^−3^	3.32 × 10^−19^	32.95
	19.6	1.83	0.366	1.70	1.28 × 10^−3^	3.32 × 10^−19^	32.95
							
Ca	193.7	9.83	1.97	15.3	7.88 × 10^−3^	2.22 × 10^−29^	39.2
	193.7	9.48	1.90	15.2	7.61 × 10^−3^	1.57 × 10^−29^	39.4
	193.7	10.0	2.00	15.4	8.02 × 10^−3^	2.43 × 10^−29^	39.1
	193.7	9.31	1.86	15.1	7.48 × 10^−3^	1.35 × 10^−29^	39.5
	193.7	9.95	1.99	15.4	7.98 × 10^−3^	2.27 × 10^−29^	39.1
	193.7	9.38	1.88	15.2	7.53 × 10^−3^	1.39 × 10^−29^	39.5

a “H^+^ added” represents the amount of protons in the volume of acid added at the start of the saturation runs. [M^2+^]_tot_ is the total concentration of Mg or Ca determined in the saturated solution. [Ins*P*_6_]_tot_ corresponds to 1/5 the amount of M^2+^, while [H^+^]_tot_ is calculated as [H^+^]_added_ plus two times the total amount of Ins*P*_6_, both according to the stoichiometry of the solid phytates previously determined. [M^2+^]_free_ and [H_2_L^10−^]_free_ correspond to free equilibrium concentrations calculated using HySS software. The p*K*_s0_ values are valid for *I* = 0.15 M NaClO_4_ and 37 °C.

**Table 2:** Calculated solubility of [Mg_5_(H_2_L)] · 22H_2_O and [Ca_5_(H_2_L)] · 16H_2_O in 0.15 M NaClO_4_ and 37.0 °C at different pH values

pH	Solubility (M)		Solubility (mg/L)	
	[Mg_5_(H_2_L)] · 22H_2_O	[Ca_5_(H_2_L)] · 16H_2_O	[Mg_5_(H_2_L)] · 22H_2_O	[Ca_5_(H_2_L)] · 16H_2_O
2.5	3.08 × 10^−2^	1.75 × 10^−3^	35969	1993
5.0	4.77 × 10^−4^	2.05 × 10^−5^	557	23.3
6.0	1.65 × 10^−4^	4.71 × 10^−6^	192	5.36
7.0	9.10 × 10^−5^	1.28 × 10^−6^	106	1.46
7.5	7.73 × 10^−5^	7.26 × 10^−7^	90.2	0.83
8.0	6.96 × 10^−5^	4.36 × 10^−7^	81.2	0.50
10.0	6.06 × 10^−5^	1.27 × 10^−7^	70.8	0.14
